# Exercise Increases Serum Fibroblast Growth Factor 21 (FGF21) Levels

**DOI:** 10.1371/journal.pone.0038022

**Published:** 2012-05-31

**Authors:** Daniel Cuevas-Ramos, Paloma Almeda-Valdés, Clara Elena Meza-Arana, Griselda Brito-Córdova, Francisco J. Gómez-Pérez, Roopa Mehta, Jorge Oseguera-Moguel, Carlos A. Aguilar-Salinas

**Affiliations:** 1 Department of Endocrinology and Metabolism, Instituto Nacional de Ciencias Medicas y Nutricion Salvador Zubiran, Mexico City, Mexico; 2 Department of Internal Medicine, Instituto Nacional de Ciencias Medicas y Nutricion Salvador Zubiran, Mexico City, Mexico; 3 Deparment of Cardiology, Instituto Nacional de Ciencias Medicas y Nutricion Salvador Zubiran, Mexico City, Mexico; Postgraduate Medical Institute & Hull York Medical School, University of Hull, United Kingdom

## Abstract

**Background:**

Fibroblast growth factor 21 (FGF21) increases glucose uptake. It is unknown if FGF21 serum levels are affected by exercise.

**Methodology/Principal Findings:**

This was a comparative longitudinal study. Anthropometric and biochemical evaluation were carried out before and after a bout of exercise and repeated after two weeks of daily supervised exercise. The study sample was composed of 60 sedentary young healthy women. The mean age was 24±3.7 years old, and the mean BMI was 21.4±7.0 kg/m^2^. The anthropometric characteristics did not change after two weeks of exercise. FGF21 levels significantly increased after two weeks of exercise (276.8 ng/l (142.8–568.6) vs. (460.8 (298.2–742.1), p<0.0001)). The delta (final–basal) log of serum FGF21, adjusted for BMI, showed a significant positive correlation with basal glucose (r = 0.23, p = 0.04), mean maximal heart rate (MHR) (r = 0.54, p<0.0001), mean METs (r = 0.40, p = 0.002), delta plasma epinephrine (r = 0.53, p<0.0001) and delta plasma FFAs (r = 0.35, p = 0.006). A stepwise linear regression model showed that glucose, MHR, METs, FFAs, and epinephrine, were factors independently associated with the increment in FGF21 after the exercise program (F = 4.32; r^2^ = 0.64, p<0.0001).

**Conclusions:**

Serum FGF21 levels significantly increased after two weeks of physical activity. This increment correlated positively with clinical parameters related to the adrenergic and lipolytic response to exercise.

**Trial Registration:**

ClinicalTrials.gov NCT01512368

## Introduction

Fibroblast growth factor 21 (FGF21) is a protein involved in glucose uptake, lipid metabolism and energy balance [1]. It is synthesized in the liver [2] and secreted following the agonist effect of free fatty acids (FFAs) on peroxisome proliferator-activated receptor alpha nuclear receptors (PPAR-alpha) [3]. It plays an important role in the adaptation to fasting in animal models. FGF21 has diverse effects including the stimulation of gluconeogenesis, fatty acid oxidation, lipolysis, ketogenesis [4], inhibition of lipogenesis [5] and blunting of the GH-signaling pathway [6]. FGF21 also increases glucose utilization (through the Akt pathway) in fat and muscle [7]. This hormone appears to improve the utilization of energy substrates (fatty acids, ketones, and glucose), and interferes with energy consuming processes (i.e. lipogenesis and growth). However, in humans under physiological conditions, its functions are still not clear. In clinical studies, a positive correlation between FGF21 plasma levels and many parameters of obesity (e.g. body mass index (BMI), waist circumference, waist to hip ratio and fat percentage) has been shown [8]). Our group has found a direct relationship between FGF21 and BMI, uric acid, fasting glucose, and the components of the metabolic syndrome [9]). We identified a direct relationship (r = 0.23, p = 0.002) between daily physical activity and serum FGF21 levels. Increased FFAs concentration may be the mechanism explaining the observed associations. Therefore, we hypothesized that serum FGF21 levels will increase after an exercise program. To confirm this theory, we carried out this longitudinal study to evaluate the impact of a two week supervised intensive physical activity program on serum FGF21 levels, in a group of young sedentary healthy women.

## Methods

The protocol for this trial and supporting CONSORT checklist are available as supporting information; see Checklist S1 and Protocol S1.

### Ethics statement

The Human Biomedical Research Institutional Committee of our Institution approved the study, and written informed consent was obtained from all subjects. All clinical investigation was conducted according to the principles expressed in the Declaration of Helsinki.

**Figure 1:**
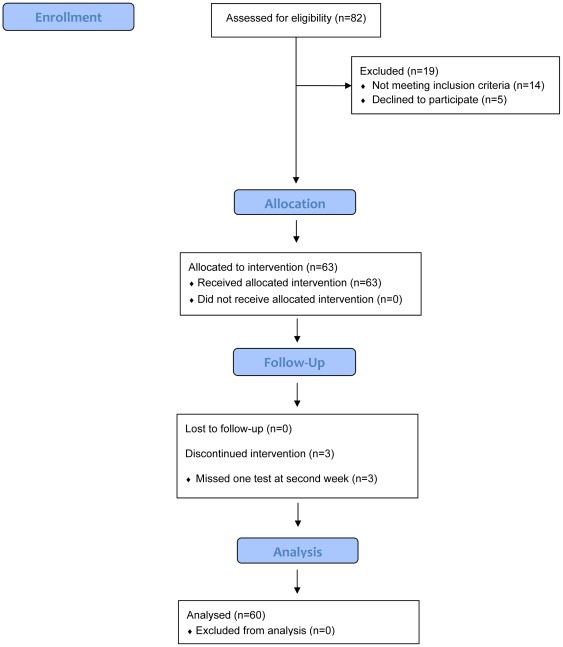
Flow chart of the study protocol.

### Study aims

**Table 1:** Clinical and biochemical parameters of the sample studied before and after two weeks of supervised physical activity (n = 60).

Variables	Before	After	*p* value
Age (yr)	24.0±3.7	-	-
SBP (mmHg)	101.3±10.0	100.4±8.8	0.38
DBP (mmHg)	65.7±6.1	64.9±6.1	0.32
BMI (kg/m^2^)	21.4±7.0	21.3±7.0	0.14
Waist circumference (cm)	74.6±8.0	74.3±7.9	0.12
Hip circumference (cm)	91.6±6.0	92.3±11.7	0.59
WHR	0.81±0.05	0.81±0.07	0.57
Fat percentage (%)	27.6±5.5	28.6±9.1	0.23
Free fat mass (gr/kg)	35.7±3.1	35.5±30.1	0.12
ALT (IU/l)	14.4±6.6	14.2±5.2	0.78
AST (IU/l)	20.1±4.9	19.0±3.9	0.05
GGT (IU/l)	13.0 (11.0–17.7)	12.0 (11.0–16.0)	0.004
Cholesterol (mg/dl)	171.5±38.5	171.9±34.2	0.90
Triglycerides (mg/dl)	101.5 (66.0–139.2)	75.5 (60.2–99.7)	0.001
HDL-C (mg/dl)	52.4±12.9	53.2±13.6	0.27
LDL-C (mg/dl)	99.2±24.7	99.8±25.9	0.75
Uric acid (mg/dl)	4.4±0.9	4.4±0.9	0.95
Serum creatinine (mg/dl)	0.66±0.11	0.65±0.09	0.30
Daily physical activity (kcal/day)*	1453 (1229–1660)	4359 (3688–4982)	<0.0001
Number of tests, n (%)			
6	-	6 (10)	-
7	-	15 (25)	-
8	-	10 (16.7)	-
9	-	29 (48.3)	-

Data are expressed as mean±SD or as median (interquartil range). NS = not significant. SBP = systolic blood pressure; DBP = diastolic blood pressure; BMI = body mass index; WHR = waist-hip-ratio; ALT = alanine aminotransfarase; AST = aspartate aminotransferase; LDL-C = low-density lipoprotein cholesterol; HDL-C = high-density lipoprotein cholesterol; GGT = gamma-glutamyltransferase.

* Daily physical activity measured with questionnaire.

The aim of the study was to evaluate the impact of a two week supervised intensive physical activity program on serum FGF21 levels, in a group of young sedentary healthy women. In order to discuss the possible mechanisms involved, the secondary outcomes of the study were to evaluate the association between FGF21 and free fatty acids (FFAs), metabolic equivalents (METs) and epinephrine, after a single bout of exercise and after the two week exercise program.

**Table 2:** Comparisons of biochemical parameters before and after supervised physical activity (n = 60).

			Parameter						
Time	N	Glucose(mg/dl)	Insulin(µU/ml)	FGF21(ng/l)	FFA(mg/dl)	Epinephrine(pg/ml)	Leptin (ng/ml)		Adiponectin(ng/ml)
Baseline	60	86.0±7.1	8.1 (5.7–11.1)	276.8 (142.8–568.6)	0.36 (0.24–0.44)	9.2 (5.9–12.1)	13.1 (8.7–21.1)		10.1 (8.0–13.4)
1 hr after first bout of exercise	60	89.8±12.2	10.8 (6.4–13.3)	237.0 (134.0–581.2)	0.34 (0.22–0.47)	10.0 (7.6–14.6)	11.6 (8.3–16.8)		9.9 (8.2–13.3)
4 hr after first bout of exercise	42	85.5±9.2	9.6 (6.3–12.9)	268.9 (192.8–582.8)	0.29 (0.15–0.41)	10.5 (5.9–14.1)	12.1 (7.8–15.9)		10.3 (8.2–13.2)
Final (after 2 weeks)	60	86.3±6.4	7.9 (5.4–10.2)	460.8 (298.2–742.1)	0.54 (0.43–0.66)	15.5 (11.3–18.8)	12.4 (10.0–16.2)		10.5 (8.0–13.2)
		***p values*** *		***p values adjusted for age, BMI, WC, glucose and insulin*** *					
Basal vs. 1 hr	60	0.177	0.621	1.00	1.00	0.005	<0.0001	0.341	
Basal vs. 4 hr	42	0.021	<0.0001	1.00	1.00	0.043	<0.0001	0.386	
Basal vs. Final	60	1.00	0.41	<0.0001	<0.001	<0.0001	0. 16	1.00	
1 hr vs. 4 hr	42	0.609	<0.0001	1.00	1.00	0.267	0.492	1.00	
1 hr vs. Final	60	0.346	0.086	<0.0001	<0.055	<0.0.04	0.882	0.909	
4 hr vs. Final	42	0.029	<0.0001	<0.0001	<0.0001	<0.0001	0.038	0.578	
Time effects	60	0.002	<0.0001	0.003	0.029	0.049	0.273	0.613	

Data are expressed as mean±SD or as median (interquartil range). FGF21 was log-transformed before analysis. NS = not significant. BMI = body mass index; WC = waist circumference; FFAs = free fatty acids.

* Bonferroni adjustment for multiple comparisons. Parameters of the repeated measures model of: Glucose: Hotteling's trace = 0.33; F = 3.29; p = 0.034; Insulin: Hotteling's trace = 1.80; F = 18.01; p<0.0001; FGF21: Hotteling's trace = 2.62; F = 22.76; p<0.0001; FFAs: Hotteling's trace = 1.58; F = 13.2; p<0.0001; Leptin: Hotteling's trace = 0.96; F = 10.93; p<0.0001; Epinephrine: Hotteling's trace = 2.29; F = 19.13; p<0.0001; Adiponectin: Hotteling's trace = 0.17; F = 1.98; p = 0.135.

### Participants

Between March 2010 and August 2011 a total of 63 sedentary young women aged 18 to 35 years old, with a BMI <30 kg/m^2^, and without any contraindication for exercising, were included in the study. Subjects were nutritionist and medical students participating in a clinical rotation at the Department of Endocrinology of the Instituto Nacional de Ciencias Médicas y Nutrición Salvador Zubirán (INCMNSZ). In addition, participants' sisters were also invited. We included 3 participants every 2 weeks in consecutive basis. We decided to study only women because the lipolytic response after exercising is higher in women than in men [10]. Furthermore, women have lower cardiovascular risk with intensive physical activity than men. Sedentary lifestyle was defined as an absence of moderate to vigorous regular physical activity during the previous 6 months [11]. We excluded subjects with the metabolic syndrome, diabetes, dyslipidemia, hypertension, asthma, and thyroid disease. In addition, women treated with fibrates, beta agonists or blockers, a past or present history of arrhythmias, murmurs, cardiomegaly, or treatment for cardiovascular diseases were excluded. Contraindications for exercise testing were also considered as exclusion criteria [12]. The flow chart of the study protocol is showed in figure 1.

**Figure 2:**
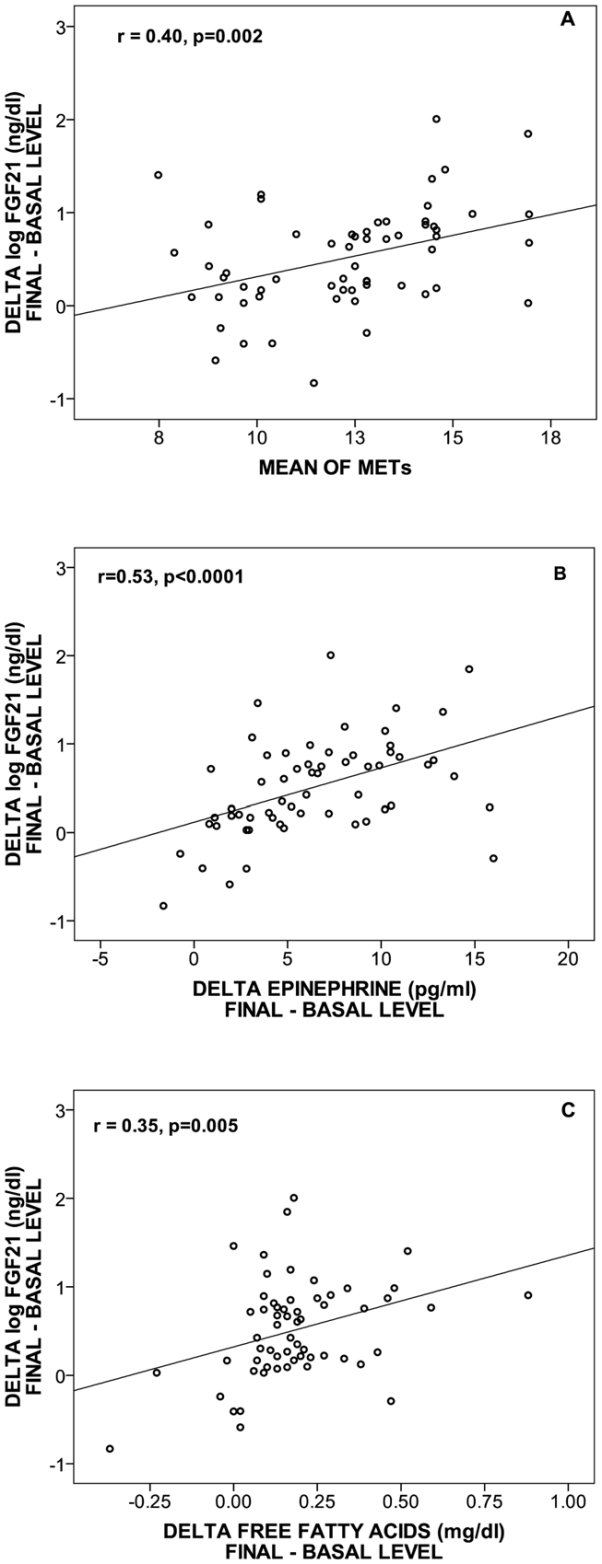
Correlation of the log delta FGF21 (final-basal) levels with METs (A), delta epinephrine (B), and delta free fatty acids (C) in 60 women after two weeks of supervised physical activity.

**Table 3:** Correlations of the DELTA log FGF21 (final-basal) with the delta and mean values of related parameters associated with physical activity, lipolysis and adrenergic response (n = 60).

Variable	DELTA FGF21	DELTA FFA	DELTA Leptin	DELTA Epinephrine	Mean RHR	Mean MHR	Mean METs
DELTA FGF21	-	0.35**	0.13	0.53**	0.43**	0.54**	0.40**
DELTA FFA	-	-	−0.13	0.40**	0.05	0.22*	0.25*
DELTA Leptin	-	-	-	0.11	0.02	−0.35	−0.19
DELTA Epinephrine	-	-	-	-	0.10	0.41**	0.18*
Mean RHR	-	-	-	-	-	0.20	0.14
Mean MHR	-	-	-	-	-	-	0.58**

FGF21 was log-transformed before analysis. All the correlations were adjusted for BMI. FGF21 = Fibroblast Growth Factor 21 (ng/l); RHR = Rest heart rate; MHR = Maximum heart rate; METs = Metabolic equivalents; FFA = Free fatty acids;

* p<0.05;

** p<0.01.

**Table 4:** Clinical and biochemical parameters stratified for tertiles of delta serum FGF21 (Final – Basal) levels.

		Tertiles of delta FGF21 of Final – Basal levels (ng/l)			
		1	2	3	
Variables	Total	<88 ng/l	88–228 ng/l	>228 ng/l	p values
N	60	20	20	20	-
Age (yr)	24.0±3.7	24.4±3.4	24.1±3.3	23.5±4.4	0.75
Basal WC (cm)	74.6±8.0	75.4±6.3	74.4±8.7	74.1±9.0	0.87
Basal BMI (kg/m^2^)	21.4±7.0	20.6±5.3	20.8±5.8	22.7±9.3	0.58
Basal Fat mass (%)	27.6±5.5	27.8±4.7	28.3±6.2	26.9±5.7	0.73
Delta FFAs (mg/dl)	0.16 (0.09–0.24)	0.10 (0.005–0.18)	0.16 (0.10–0.24)	0.20(0.13–0.38)	0.01
Delta Epi (pg/ml)	6.05 (3.0–9.7)	2.86 (1.3–6.6)	7.0 (5.1–9.7)	6.9 (4.1–10.4)	0.008
Delta Leptin (ng/ml)	−0.30 (−5.4–3.1)	−0.74 (−5.9–1.2)	0.66 (−5.6–5.4)	0.94 (−3.5–3.1)	0.32
Mean RHR (bpm)	89.5±11.7	86.7±4.2	88.6±7.6	93.2±7.9	0.02
Mean MHR (bpm)	176.2±12.8	165.2±7.5	181.3±12.3	182.1±10.5	<0.0001
Mean METs	12.2±2.4	10.9±2.1	13.2±1.7	12.5±2.7	0.008
Mean Resting SBP	99.3±5.7	100.8±5.5	98.2±5.6	98.8±5.9	0.32
Mean Resting DBP	65.7±3.2	65.5±3.4	65.8±3.4	65.7±3.0	0.93
Mean Max SBP	127.1±11.02	126.7±10.6	131.2±10.8	133.3±10.5	0.05
Mean Max DBP	72.7±5.7	71.2±5.2	75.6±5.6	76.3±5.4	0.01

Data are expressed as mean±SD or as median (interquartil range). FGF21 was log-transformed before analysis. P values obtain from one-way ANOVA or Kruskall-Wallis tests as appropriate. NS = not significant. WC = waist circumference; BMI = body mass index; FFAs = free fatty acids; Epi = epinephrine; BPM = beats per minute; RHR = resting heart rest; MHR = maximum heart rate; METs = metabolic equivalents. SBP = systolic blood pressure; DBP = diastolic blood pressure. delta = Final – Basal levels. Resting = value obtained before each test. Max = maximum value obtained during each test. Mean = sum of values/number of tests.

**Table 5:** Multiple stepwise regression analysis of variables independently associated with the delta log serum FGF21 levels (model 1) and free fatty acids (model 2).

Model 1				
Independent variables	β	Standardized β	t	p value
Basal Glucose	0.016	0.208	2.732	0.009
Delta FFA	1.018	0.340	2.823	0.007
Delta Epinephrine	13.88	0.269	2.248	0.030
Mean MHR	0.021	0.490	3.084	0.004
Mean METs	0.089	0.389	3.217	0.002

Model 2				
Independent variables	β	Standardized β	t	p value
Insulin	−0.016	−0.376	−2.24	0.03
Time of exercise	−0.046	−0.358	−2.52	0.01
Delta Epinephrine	0.014	0.306	2.08	0.04
Delta FGF21	0.170	0.510	3.45	0.001

The analyses also included age, duration of exercise, creatinine, waist to hip ratio, fat percentage, body mass index, mean resting heart rate, systolic and diastolic blood pressure, which were all excluded in the final models. FFAs = free fatty acids. MHR = maximum heart rate. Delta = Final – Basal levels. Parameters of model 1: F = 3.48; r^2^ = 0.54, p<0.001. Parameters of model 2: F = 3.4; r^2^ = 0.44, p = 0.001. Maximum = maximum value obtained during each test. Mean = sum of values/number of tests.

### Study protocol

This was an interventional study of one group and every subject was used as its own control. All subjects underwent a basal and final physical examination. The routine biochemical analyses were carried out after overnight fasting between 8 to 12 h. In the initial visit, patients were instructed not to alter their diet, lifestyle and level of physical activity. Basal daily physical activity was evaluated with a questionnaire developed by Tremblay et al and validated in the Mexican population [13]. The questionnaire quantifies physical activity level (kcal/day or in kJ if kcal are multiplied by 4.1855) throughout a 24hr-day as previously described [9]. In brief, every subject answered three questionnaires distributed over two labor days and one day of the weekend. These results were added together and the average kcal/day was obtained. The participants answered the questionnaires at the beginning and end of the study.

A treadmill exercise test (following the Bruce's protocol) was carried out five times a week (from Monday to Friday) for two weeks. The Bruce protocol is a multistage, well standardized test, which includes electrocardiographic monitoring during exercise [12]). The protocol has seven stages, each lasting three minutes, resulting in 21 minutes' of exercise for a complete test. In stage 1, patients walk at 1.7 mph (2.7 km) up a 10% incline. Energy expenditure is estimated to be 4.8 metabolic equivalents (METs) during this stage. The speed and inclination is increased in each stage. From the second to the seventh stage, the speed increases from 2.5 mph (4.0 km/hr) to 6.0 mph (9.6 km/hr), and inclination from 12 to 22%; METs increase from 7.0 to 22.0, respectively. Assessment of workload is measured by METs which is an indirect reflection of oxygen consumption and energy use. One MET is 3.5 ml oxygen/kg per minute, which is the oxygen consumption of an average individual at rest. To carry out the activities of daily living an exercise intensity of at least 5 METs is required [12]. By convention, the maximum predicted heart rate is calculated as 220 (210 for women) minus the subject's age. A satisfactory heart rate response is achieved on reaching 85% of the maximum predicted heart rate [12]. Therefore, we maintained the heart rate above an intensity of 85% for at least 15 minutes in each test. The first exercise test was carried out in the morning between 7–8:30 AM. Participants were given the option of appointments between 7:00 to 8:30 AM or between midday and 14 hrs depending on the availability of the treadmill and to favor adherence for subsequent tests. Although most participants came at same hour every day, sometimes they accomplished with the study running in the other schedule. We considered two criteria for stopping the test: 1) when a participant reached her maximum predicted heart rate to reduce the probability of adverse events; and 2) because of fatigue. Systolic (SBP) and diastolic (DBP) blood pressure were measured before (at rest) and during (at two minutes intervals) in each test.

The study had a duration of 2 weeks with a total of 9 tests. Additional physical activity was prohibited. Participants with one missing test in the second week of exercising were eliminated from the study (n = 3, 4.7%).

Glucose, insulin, FFAs, FGF21, and leptin plasma levels were measured before and at 1 and 4 hours after the first exercise test to evaluate the acute impact of exercise. This sampling schedule was based on the effects of elevated FFAs on FGF21 in humans 14]. During this time period, participants remained fasting.

### Biochemical and anthropometric measurements

The Department of Endocrinology and Metabolism at the INCMNSZ performed all biochemical laboratory measurements using standardized procedures. The measurements were performed with commercially available standardized methods. Glucose was measured using the glucose oxidase method (Boehringer Mannheim, Germany), serum total cholesterol and triglycerides were measured using an enzymatic method (Beckman), HDL and LDL cholesterol levels were measured after precipitation using phosphotungstic acid and Mg2^+^ (Beckman), plasma insulin was determined by monoclonal assay (MEIA, Abbott Laboratories), and creatinine and uric acid were measured using commercially available kit (Beckman Coulter, Inc.). All equipment was regularly calibrated using reference samples provided by the manufacturer. A human FGF21 ELISA kit was used (BioVendor Laboratory Medicine, Modrice, Czech Republic) following the procedure described previously [9]. Serum adiponectin and leptin (Millipore, Mexico), free fatty acids (Wako Chemicals, USA), and adrenaline (MP Biomedicals, USA) were determined by a sandwich ELISA kit. Anthropometric measurements were carried out after participants removed their shoes and upper garments. Body weight and fat was quantified with the UM-026 Tanita Body Composition Analyzer (Tanita, Tokyo). All subjects were instructed to stand in the centre of the scale during weight assessment. Height was obtained using the floor scale's stadiometre, again with the patient standing on the central part of the scale. Height was measured to the nearest 0.5 cm. BMI was calculated as weight (kg) divided by height (m^2^). Abdominal circumference was measured to the nearest 0.1 cm at the level of the greatest frontal extension of the abdomen between the bottom of the rib cage and the top of the iliac crest.

### Statistical analysis

The sample size was calculated using the formula for means for two-tailed comparisons. According to our previous report [9], we expected a minimal change of 80 ng/l of FGF21 after two weeks of physical activity. Using a SD of 160 ng/l with an alfa of 0.05 and a study power of 80%, a total of 60 subjects were calculated. All participants were included in each statistical analysis. Normally distributed data, determined using Kolmogorov-Smirnov test, were expressed as means and standard deviation (±SD), whereas variables with a skewed distribution were reported as median (interquartile range) and log transformed to approximate normality before analyses. Chi Square, Student's unpaired t test, Wilcoxon signed rank test or Mann-Whitney U test was used as appropriate for comparison of measurements before and after physical activity. Homogeneity of variance was evaluated with Levene's test. For data collected at multiple time points we used repeated measures analysis with effects for time. Sphericity was evaluated with Mauchly's test. When necessary, correction of significance with Greenhouse-Geisser or Huynh-Feldt epsilon was applied. Pairwaise comparisons between time points were done based on estimated marginal means. Bonferroni correction was used for adjustment for multiple comparisons. Correlation coefficients between FGF21 and continuous variables were evaluated in all participants, and were calculated using the Spearman's rho, Pearson's r tests or using partial correlation analysis when adjusted for BMI. Kruskall-Wallis test or one-way ANOVA were used for comparing continuous variables of patients stratified by tertiles of serum levels of FGF21. To evaluate the effect of exercise on clinical and biochemical parameters, we used the difference between final – basal levels (as indicated with “delta”). “Resting” refers to the value obtained before each test. “Maximum” refers to the highest value obtained during each test. Mean refers to the sum of values/number of tests. We calculated the mean of resting and maximum heart rate, systolic and diastolic blood pressure as specified. A stepwise linear regression model was used to examine the impact of variables in delta log serum FGF21 levels (model 1) and delta FFAs (model 2). Homogeneity and linearity were confirmed through the pattern of studentized residuals plotted against adjusted predicted values. The variables selected for the regression analyses were those that correlated significantly with serum FGF21 or FFAs and those that have been shown to be associated with plasma levels of these proteins [4], [9]. All reported p values are based on two-sided tests considering ≤0.05 as significant. All analyses were performed with SPSS 17.0 (Chicago, IL).

## Results

A total of 60 women with a mean age of 24.0±3.7 years and a mean BMI of 21.4±7.0 kg/m2 were evaluated. The baseline and final characteristics of the study population are shown in Table 1. The anthropometric data of participants did not change after two weeks of physical activity. Significant reductions in triglyceride and gamma glutamyltransferase (GGT) plasma levels were observed following the exercise program (Table 1). The median (interquartil range) of daily physical activity at baseline quantified with the questionnaire was 1453.2 (1229.4–1660.8) kcal/day. After two weeks, an expected and significant increment in kcal/day was identified (4359.6 (3688.3–4982.5), p<0.0001).

Almost half of the participants completed the 9 scheduled tests. The remaining 16.7%, 25%, and 10% completed 8, 7 and 6 tests, respectively (Table 1). The mean duration of the exercise tests was 14.2±1.4 minutes per session. The majority of participants reached the stage 4 of the Bruce's protocol, with a mean METs consumption of 12.2±2.4. Tolerance to exercise increased during the study. The mean duration of exercise in the first week (tests 1 to 5) was of 14.0±1.6 minutes per session. This time increased significantly in the second week (14.5±1.5 min, p = 0.002). Similarly, the METs consumed in the second week were greater (12.2±2.3) than in the first (11.3±2.5, p<0.0001).

At baseline, log serum FGF21 levels correlated positively with FFA (r = 0.12, p = 0.03), fasting glucose (r = 0.29, p = 0.04) and physical activity (r = 0.16, p = 0.05), and negatively with adiponectin (r = −0.27, p = 0.03), and creatinine (r = −0.25, p = 0.04). FFAs correlated positively with fasting glucose (r = 0.30, p = 0.02) and negatively with baseline insulin (r = −0.31, p = 0.01). No significant correlation was identified between log FGF21 and fasting insulin (r = 0.17, p = 0.17).

### Acute effects of exercise on FGF21

The baseline levels of glucose, insulin, FGF21, FFA, epinephrine, leptin and adiponectin were compared with their corresponding levels at one and four hours following the first exercise session (table 2). At one hour, no significant changes in glucose and insulin were documented. At the fourth hour, glucose was reduced to a level below baseline (p = 0.021) and insulin showed a significant increment in comparison with the baseline levels (p = 0.0001). A significant increment in adrenaline and a significant reduction in leptin was also documented (all p<0.05). However, acute exercise did not change the serum levels of FFAs, FGF21, and adiponectin (table 2).

### Change in FGF21 levels after two weeks of exercising

After two weeks of exercising there was a significant increase in serum FGF21 (276.8 (142.8–568.6) vs. 460.8 (298.2–742.1) ng/l, p<0.0001), and FFAs values (0.36 (0.24–0.44) vs. 0.54 (0.43–0.66) mg/dl, p<0.001). No significant changes were identified in serum leptin or adiponectin levels.

The delta of log serum FGF21, had a significant positive correlation with delta epinephrine (r = 0.53, p<0.0001), METs consumption (r = 0.40, p<0.0001), and delta FFA levels (r = 0.35, p<0.0001, figure 2). Similar results were obtained after adjustment for BMI. The mean resting (r = 0.43, p<0.0001) and maximum (r = 0.54, p<0.0001) heart rate also correlated positively with delta log FGF21 (table 3). Additionally, subjects in the second and third tertile of the delta log FGF21 had a higher level of mean resting heart rate (RHR, p = 0.02), mean maximum heart rate (MHR, p<0.0001), mean METs (p = 0.008), delta FFAs (p = 0.01), delta epinephrine (p = 0.008), and mean maximum systolic (p = 0.05) and diastolic (p = 0.01) blood pressure in comparison with the first tertile (table 4). No significant association was identified with anthropometric parameters, resting blood pressure and delta leptin levels with tertiles of delta log FGF21.

Delta FFAs correlated positively with delta epinephrine, heart rate and METs (table 3). The mean maximum DBP (r = 0.25, p = 0.05), and SBP (r = 0.26, p = 0.03) were also significantly associated with delta FFAs. Interestingly, the mean time per test (r = −0.34, p = 0.007) correlated negatively with the increment in FFAs.

Stepwise linear regression model showed that baseline fasting glucose, delta FFAs, delta epinephrine, MHR, and METs were factors independently associated with the increment in serum FGF21 levels after exercise (F = 3.48; r^2^ = 0.54, p<0.0001, table 5). In a second model we evaluated the determinants of delta FFAs. Interestingly, basal insulin, mean duration per exercise test, delta epinephrine, and delta log FGF21 were factors independently related to the increment in serum FFAs levels (F = 3.4; r^2^ = 0.44, p = 0.001, table 5).

## Discussion

FGF21 plays an important role in carbohydrate and lipid metabolism, and energy balance [1)]. However, in humans under physiological conditions, its functions are not clear. Our group identified that FGF21 levels are associated with the amount of physical activity recorded using a validated questionnaire [9]. Here, we investigate if a period of supervised exercise altered serum FGF21 levels in healthy adults. Results showed that serum FGF21 levels significant increased after two weeks of daily physical activity, but no change was observed after a single bout of exercise. The increment in FGF21 showed a positive correlation with the changes in serum FFA levels. Interestingly, METs, RHR, MHR and delta epinephrine (markers of adrenergic activation) correlated positively with the increase in FGF21.

Exercise increases lipolysis through the agonist effect of epinephrine on beta 3 adrenergic receptors in adipose tissue [15]–[18]. The amount of exercise considered in our protocol induced a significant adrenergic response measured clinically (with heart rate and blood pressure) and biochemically (with epinephrine). The adrenergic response is greater in women who are not physically fit. These characteristics allow us to evaluate with certainty the acute and medium-term effects of exercise on FGF21 serum levels.

Baseline fasting glycemia, delta FFAs, epinephrine, heart rate and METs were associated with an exercise –induced increment in FGF21 levels. The FFA response may be the main signal for increasing FGF21 concentrations. The FGF21 gene has a PPAR alpha response element that is activated by the complex FFA/PPAR alpha/RXR [19], [20]. The central role of PPAR alpha activation is demonstrated by the almost complete elimination of the effects of fasting on FGF21 expression in the PPAR alpha knockout mouse [21], and the increment of hormone in response to an IV infusion of fatty acids [14]. However, in order to become a biologically relevant signal for increasing the expression of the FGF21 gene, supraphysiological concentrations of FFAs are needed [22]. This phenomenon may explain the increased FGF21 concentrations reported in prolonged fasting [3], lactation [23], and growth hormone therapy [24], conditions associated with remarkably high FFA plasma levels. The same observation may explain the weak correlation observed between FGF21 and baseline serum FFAs (r = 0.12, p = 0.03) reported in both our study and by other groups [4], [23]. Thus, it is likely that chronic exposition to increased FFA concentrations may have a stronger association with FGF21 serum levels than fasting FFA. This proposal may explain the strong association reported between serum FGF21 levels and liver fat content [25]. On the other hand, another determinant of the change in FGF21 levels in our study was baseline fasting glycemia. This association is explained by the presence of a carbohydrate response element binding protein (ChREBP) response element in the FGF21 gene [22]. Previous reports have shown that fasting glycemia is associated with FGF21 serum levels [9], [26]. Our report provides confirmatory evidence and shows that the prominent role of glycemia continues under conditions of stimulated lipolysis. The other predictors of the change in FGF21 levels (i.e. epinephrine, heart rate and METs) are markers of the exercise induced adrenergic response and the subsequent increment of FFAs. The variables discussed above determined more than 50% of the increment in FGF21 after two weeks of physical activity. Thus, a large percentage of the variability in the FGF21 response to exercise can be explained by the variables evaluated here.

Exercise improves glucose disposal by increasing insulin action and by activating the AMPK pathways, causing GLUT4 translocation to the muscle cell surface and glucose uptake [27]. The FGF21 response to exercise could be involved in the beneficial effects of increased physical activity in lipid and carbohydrate utilization, in healthy and obese individuals. The increment in serum FGF21 may aim to decrease the level of FFAs through the inhibition of lipolysis [28]. In addition, both chronic exercise and FGF21 induce expression of the peroxisome proliferator-activated receptor c coactivator protein-1a (PGC1alpha), a transcriptional coactivator protein that interacts with several different DNA-binding proteins resulting in increased gluconeogenesis, fatty acid oxidation, and ketogenesis [4], [6], [29]. These actions lower serum FFAs concentrations and may protect against the ectopic deposit of lipids in the liver and the muscle. Lipotoxicity plays a major role in the pathogenesis of the obesity-related metabolic complications. This interpretation is in agreement with the increased FGF21 levels reported in patients with the metabolic syndrome, obesity, and impaired glucose tolerance [8], [9], [26]. Probably, the increment of FGF21 is a mechanism to counteract the chronic exposition of increased FFAs plasma levels and lipotoxicity. Exercise may reinforce this compensatory pathway. However, the FGF21 increment only happens after a chronic stimulus capable of inducing adrenergic activation. A single bout of intense exercise did not alter FGF21 serum levels. A potential explanation for this finding is the increased insulin response observed after a single session of exercise. Insulin inhibits lipolysis and blocks the main mechanism by which exercise stimulates FGF21 secretion (i.e. increased FFAs plasma levels). Thus, increased FGF21serum levels may be an additional mechanism by which exercise improves carbohydrate and lipid metabolism in the medium term.

Some limitations of our study should be recognized. We cannot fully control other potential sources of physical activity. We fully relied on the adherence of the participants to our recommendations. However, the second evaluation with the questionnaire suggests that participants considerably increased the kcal/day consumed because of the study. In addition, the results may not be applicable to men. Finally, we did not register the caloric consumption during the study. However, no major changes in BMI and body composition were observed.

In summary, serum FGF21 increased significantly after two weeks of daily physical activity. This increment is related to clinical and biochemical parameters of adrenergic response and serum levels of FFAs.

## Supporting Information

Checklist S1 CONSORT Checklist.
(PDF)

Protocol S1 Trial Protocol.
(PDF)
