# Supplementary material for: Exercise Increases Serum Fibroblast Growth Factor 21 (FGF21) Levels
Source: PLoS One. 2012 May 31;7(5):e38022. doi: 10.1371/journal.pone.0038022 (PMC3365112; doi:10.1371/journal.pone.0038022)
Supplement: Protocol S1 — Trial Protocol. (PDF) [file pone.0038022.s002.pdf]

COMITÉ INSTITUCIONAL DE INVESTIGACIÓN BIOMÉDICA EN HUMANOS

FORMATO DE EVALUACIÓN DE PROYECTO DE INVESTIGACIÓN

Colocar cursor dentro del área blanca para llenar la información (NO utilizar áreas sombreadas)

No. de registro CIIBH: 173

1. Title

Response of serum fibroblast growth factor 21 (FGF21) levels to exercise program

2. Researchers

2a. Identification

Name	Institutional position	Research position	Telephone (ext.)	E-mail
Daniel Cuevas-Ramos	Consultant, Clinical Researcher	Principal researcher	54870900 (2407)	shiverqks@hotmail.com
Paloma Almeda-Valdés	Consultant, Clinical Researcher	Coinvestigator	54870900 (2407)	palomaalmeda@yahoo.com
Clara E. Meza-Arana	Nutritionist	Coinvestigator	54870900 (2407)	claraelena88@hotmail.com
Griselda Brito-Córdova	Nutritionist	Coinvestigtor	54870900 (2407)	grisbrito@hotmail.com
Fco. J Gómez-Pérez	Chief, Department of Endocrinology	Coinvestigator	54870900 (2407)	fcojgomezp@aol.com
Jorge Oseguera-Moguel	Chief, Departament of Cardiology	Coinvestigator	54870900 (2405)	droseguera@intermedik.com.mx
Carlos Aguilar-Salinas	Subdirector, Department of Endocrinology	Coinvestigator	54870900 (2407)	caguilarsalinas@yahoo.com

3. Institutions participating in the study

Instituto Nacional de Ciencias Médicas y Nutrición Salvador Zubirán (INCMNSZ), Vasco de Quiroga 15, Sección XVI, CP14000, Tlalpan, Mexico City, Mexico

4. Funding

Department of Endocrinology, INCMNSZ
Science and Technology National Council (CONACYT)

4b. Monetary income to researcher related with the study protocol

None

5. Background

Introduction

The fibroblast growth factor 21 (FGF21) is a recent described member of the FGFs family. It is particularly expressed in liver (1) and adipose tissue (2). Its main function is to increment glucose uptake in adipose tissue (3). When is overexpressed in transgenic mice, protects animals to diet induced obesity. In addition, the subcutaneous adminsitration to mice (3) and rhesus monkeys with diabetes (4) decreased glucose and triglyceride levels. *In vitro* and *in vivo* studies have shown a significant association between FGF21 with glucose and insulin metabolism. The administration in diabetic mice also produces a significant reduction in postprandial glucose and insulin (3).

Badman *et al.* (5) e Inagaki *et al.* (6) showed that FGF21 expression in the liver of fasting mice was activated by PPAR-alfa nuclear receptor. The endogenous ligand of PPAR-alfa are fatty acids. The expression of PPAR-alfa in liver also induces higher activity of enzymes related with fatty acid oxidation and acetyl-CoA formation. The result is the increment of ketone bodies as a additional source of energy. From the liver, ketone bodies are released for future utilization in other tissues, particularly the brain. As reported Badman *et al* (5) and Inagaky *et al* (6), FGF-21 increased fatty acids oxidation and induce the metabolic process to ketone body formation. In addition, a significant increment in FGF21 transcription was induced with fasting, and it was absent in knock-out mice without PPAR-alpha. These observations, conclude the authors, link FGF21 and PPAR-alpha in one signaling pathway.

Definition of the study problem

Clinical studies in humans have shown inconsistent results regarding the role of FGF21 in metabolism. A positive and significant correlation have been reported between FGF21 and cardiovascular parameters such as BMI, waist circumference, waist to hip ratio, and fat percentage (2,7). However, similar correlations have not been found in patients with early onset type 2 diabetes (8). While some reports related FGF21 with insulin sensitivity (3,9), other studies did not confirmed such association using hyperglycemic clamp (10). Although the current information supports the role of FGF21 as metabolic regulator inducing glucose uptake (3), and fatty acids oxidation (5,6), further research is necessary to clarify its function, regulation and clinical relevance in humans. Currently, a direct relationship between glucose and FGF21 has been reported (7,8). In addition, the serum FGF21 levels are higher in patients with metabolic syndrome than without it (4,7). Nowadays, the associated mechanism that explains this increment is not well understood.

Justification of the study

Recently, our group identified a significant association between BMI, fasting glucose, uric acid and physical activity with FGF21 (7). Associated with the higher of level of FGF21 in insulin resistance states (4,7,8), it could be suspected that circumstances with higher level of free fatty acids (FFAs) induce activity of PPAR-alpha in liver and the expression and synthesis of FGF21 is incremented. Sedentary lifestyle is frequently related with overweight, obesity, and metabolic syndrome. Usually, these abnormalities are related with higher serum level of FFAs. Therefore, we consider justify evaluating the effect of other clinical circumstances that induce lipolysis and increase FFAs level on serum FGF21. Using a better methodological clinical design, we propose a study to confirm the impact of physical activity on FGF21. Moreover, the measurement of FFAs could also allow us to identify the possible mechanism related.

6a. Hypothesis

Serum FGF21 levels will increase after a supervised intensive exercise program.

6b. Study aims

a. Primary aim

1. Evaluate the impact of two weeks of supervised intensive physical activity program on serum FGF21 levels, in a group of young sedentary healthy women.

a. Secondary aims

1. Evaluate the association between FGF21 with:
a. Serum free fatty acids (FFAs) level,
b. Metabolic equivalents (METs),
c. Epinephrine,
d. Glucose and insulin,
e. Anthropometric parameters
f. Leptin and total adiponectin

7. Methodology

Study design

Longitudinal, comparative, clinical intervention study to evaluate the possible change on serum FGF21 levels after a single bout and after two weeks of exercising.

Description of intervention

Physical activity

Selected women will be invited to a treadmill test following the Bruce's protocol. The FGF21 level will be measured at the beginning (baseline), after 1 hour and 4 hours of the first test, and after two weeks of daily activity (final). We will study only women because the lipolytic response after exercising is higher in women than in men (11), and have lower cardiovascular risk with intensive physical activity. The treadmill test will be done every day during two weeks (except weekends). The schedule will be determined according the availability of the treadmill in the Department of Cardiology of this Institution.

Bruce's protocol

The Bruce's protocol is a multistage, well standardized test, including monitoring the cardiac function with electrocardiogram during exercise (12). The protocol has seven stages, each lasting three minutes, resulting in 21 minutes' exercise for a complete test. In stage 1, patients walk at 1.7 mph (2.7 km) up a 10% incline. Energy expenditure is estimated to be 4.8 metabolic equivalents (METs) during this stage. The speed and inclination increased in each stage. From the second to the seventh stage, the speed increases from 2.5 mph (4.0 km/hr) to 6.0 mph (9.6 km/hr), and inclination from 12 to 22%. METs increases from 7.0 to 22.0, respectively. Assessment of workload is measured by metabolic equivalents (METs) which are an indirect reflection of oxygen consumption and energy use. One MET is 3.5 ml oxygen/kg per minute, which is the oxygen consumption of an average individual at rest. To carry out the activities of daily living exercise intensity of at least 5 METs is required (12). By convention, the maximum predicted heart rate is calculated as 220 (210 for women) minus the subject's age. A satisfactory heart rate response is achieved on reaching 85% of the maximum predicted heart rate (12). Therefore, we will maintain the heart rate above the 85% at least 15 minutes in each test. In the following table the Bruce's protocol is described:

**Bruce's Protocol**					
Phase	*Speed (millas/hr)*	*Speed (km/hr)*	*Inclination (%)*	*METs*	*Time (minutes)*
1	1.7	2.7	10	4.7	3
2	2.5	4.0	12	7.0	3
3	3.4	5.4	14	10.1	3
4	4.2	6.7	16	12.9	3
5	5.0	8.0	18	16.0	3
6	5.5	8.8	20	19.0	3
7	6.0	9.6	22	22.0	3

METs are used to quantify the intensity of physical activity in one person. The degree of physical activity can be classified in three levels:

Low intensity: 1.1 - 2.9 METs

Moderate intensity: 3.0 – 5.9 METs

Vigorous intensity: 6.0 or more METs

The physical activity planned in the participants will be of vigorous intensity to induce a substantial adrenergic response, lipolytic effect and increment of FFAs in blood. However, the treadmill test will be stopped if any of the following circumstances is present:

a. Fatigue: when the participant ask to stop the test because of fatigue or muscle pain.
b. Maximum heart rate: the test will be stopped if the participant reach its maximum heart rate defined as 210-age.

Clinical evaluation

a) A complete clinical history and exploration will be developed in each participant to confirm inclusion and discard exclusion criteria (see below).
b) In addition, a standardized nutritionist will be measuring weight, height, waist and hip circumferences at the beginning and the end of the study. Fat percentage and free fat mass will also be measured using a UM-026 Tanita Body Composition Analyzer (Tanita, Tokyo).

Biochemical evaluation

The baseline and final samples will be taken after 8 to 12 hour fasting. The following parameters will be measured:

a) Glucose, insulin, uric acid, creatinine, total colesterol, triglycerides, HDL-c, LDL-c, GGT, and liver tests.
b) FGF21, leptin and total adiponectin.
c) Free fatty acids.
d) Epinephrine

After the first hour and fourth hour of the first treadmill test, the following parameters will be measured:

a) Glucose and insulin
b) FGF21, leptin and adiponectin
c) Free fatty acids
d) Epinephrine

Serum samples will be preserved at -80° C until its analysis. Additionally, women that could be pregnant because of a menstrual period delay will be evaluated with serum beta subunit of human chorionic gonadotropin.

Sample size

Sample size for FGF21

We expected a minimum delta of 80 ng/L of FGF21 between the baseline and final evaluations. The SD of FGF21 in Mexican population with and wihtout metabolic síndrome was of 160 ng/L (7). Considering an alpha of 0.05 (two sided) and beta of 0.20, with study power of 80%, we obtained a simple size of 63 subjects. The formula and calculations is described as follows:

$$N = 2S^2 (Z\alpha+Z\beta)^2 / \Delta^2$$

$$N = 2(160)^2 (1.96+0.84)^2 / \Delta^2$$

$$N = 96{,}800 (7.84) / 80^2$$

N = 63 subjects (only one study group).

8. Methodology: Selection Criteria

Inclusion criteria

1. Women
2. Age between 18 to 35 years
3. Written informed consent signed.

Exclusion criteria

1. Individuals with the following circumstances will be excluded:
   a. Diabetes (any type), treatment with insulin or glucose lowering drugs (i.e., metformin).
   b. History of cardiovascular disease such as heart failure, ischemic heart disease or stroke.
   c. Liver failure or chirrosis
   d. Kidney failure (acute or chronic)
   e. Peripheral vascular insuficiency.
   f. Chronic disease such as HIV, lupus, viral hepatitis, etc.
   g. Acute infection (i.e., flu, diarrhea).
2. Metabolic syndrome defined by ATP III Criteria (13).
3. Treatment for:
   a. Dyslipidemia (i.e, fibrates or statins)
   b. Asthma (i.e., beta agonists)
   c. Hypertension (i.e., beta blockers)
4. Contraindication for treadmill test (12):
   a. Ischemic heart disease
   b. Angina
   c. Heart failure
   d. Acute myocarditis or pericarditis

e. Acute systemic infection
f. Deep venous thrombosis
g. Uncontrolled hypertension (systolic >220 mmHg or diastolic > 120 mmHg)
h. Severe aortic stenosis
i. Hypertrophic cardiomiopathy
j. Arrhythmia
k. Aneurysm
l. Recent aortic surgery

5. Pregnancy or lactation

Elimination Criteria

1. Confirmed pregnancy throughout study
2. Missed any treadmill test at second week
3. Physical injury during study

9. Methodology: Outcomes and variables

Variables and definitions

Treadmill test using Bruce's protocol

Supervised physical activity will be developed following the Bruce's protocol as described above (12). We will use the METs as ordinal variable to quantify the energy consumption throughout study.

Anthropometry

Anthropometric measurements will be done after participants removed their shoes and upper garments. Body weight and fat percentage will be quantified with the UM-026 Tanita Body Composition Analyzer (Tanita, Tokyo). All subjects will be instructed to stand in the centre of the scale during weight assessment. Height will be obtained using a floor scale's stadiometre, again with the patient standing on the central part of the scale. Height will be measured to the nearest 0.5 cm. BMI will be calculated as weight (kg) divided by height (m^2). Abdominal circumference will be measured to the nearest 0.1 cm at the level of the greatest frontal extension of the abdomen between the bottom of the rib cage and the top of the iliac crest. All measurements will be done after 8 to 12 hour of fasting.

During clinical evaluation a standarized nutritionist will measure:

Parameter	***Measurement***	***Variable type***	***Definition***
Weight	kg.	Quantitative continuous	Measurement in a daily calibrated scale.
Height	Meters	Quantitative continuous	Measurement in a floor scale's stadimetre for adults.
Blood pressure	mmHg	Quantitative continuous	After resting 5 min. Every 2 minutes during treadmill test.
			Sphygmomanometer with mercury manometer and well sized cuff.
Waist	cm.	Quantitative continuous	Measuring tape adjusted for millimeters (description above).
Hip	cm.	Quantitative continuous	Measuring tape adjusted for millimeters (description above).
Fat percentage	%	Quantitative continuous	Tanita analyzer (description above).

Using these parameters the following indexes will be calculated:

a) Body mass index (BMI): Weight (kg) / Height^2
b) Hip to waist ratio (WHR): Waist (cm.) / Height (cm.)

Biochemical evaluations

The laboratory of the Department of Endocrinology and Metabolism of the Instituto Nacional de Ciencias Médicas y Nutrición Salvador Zubirán (INCMNSZ) will measure the biochemical parameters following standardized procedures. Commercial assays will be used. All subjects will be evalauted after 8 to 12 hours of fasting. Creatinine, uric acid, fasting glucose, lipid profile, liver tests and GGT will be measured using enzymatic assay by Beckman Coulter, Inc. Fasting insulin will be measured using a monoclonal assay (MEIA, Abbott Laboratories). All equipment is regularly calibrated using reference samples provided by the manufacturer. A human FGF21 ELISA kit will be used (BioVendor Laboratory Medicine, Modrice, Czech Republic) following the procedure described previously (7). This assay does not

have cross reaction with other human FGFs, adiponectin or leptin. Serum adiponectin and leptin (Millipore, Mexico), free fatty acids (Wako Chemicals, USA), and adrenaline (MP Biomedicals, USA) will be determined by sandwich ELISA kit.

Frequency of measurements (clinical and biochemical)

a) Baseline after 8 to 12 hour of fasting.
b) After first hour and forth hour of the first treadmill test.
c) Final measurement after two weeks of physical activity.

Success and failure criteria

We will consider success with the increment of serum FGF21 levels after a single bout and/or after two weeks of physical activity. In the same way, we will consider failure the absence of any change in the protein.

Statistical analyses strategy

Normally distributed data will be determined using Kolmogorov-Smirnov test and will be expressed as means and standard deviation (±SD), whereas variables with a skewed distribution will be reported as median (interquartile range) and log transformed to approximate normality before analyses. Chi Square, Student's unpaired t test, Wilcoxon signed rank test or Mann-Whitney U test will be used as appropriate for comparison between groups. Homogeneity of variance will be evaluated with Levene's test. Correlation coefficients between FGF21 and continuous variables will be evaluated in all participants, and will be calculated using the Spearman's, Pearson's r tests or using partial correlation analysis when adjusted for BMI. Kruskall-Wallis test or one-way ANOVA will be used for comparing continuous variables of patients stratified by tertiles of serum levels of FGF21. To evaluate the effect of exercise on clinical and biochemical parameters, we will be use the difference between final – basal levels (or "delta"). We will calculate the mean of resting and maximum values and will be defined as the sum of values / number of tests. We will calculate the mean of resting and maximum heart rate, systolic and diastolic blood pressure as specified. Stepwise linear regression model will be used to examine the impact of variables in delta log serum FGF21 levels (model 1) and delta FFAs (model 2). The variables selected into the regression analyses will be those that correlate significantly with serum FGF21 or FFAs and those that have been associated with plasma levels of these proteins (4, 13). All reported p values will be based on two-sided tests considering ≤0.05 as significant. All analyses will be performed with SPSS (Chicago, IL).

10. Risk and benefits of the study

Studies with risk

Throughout this study, the risk of an adverse event during the simple test is practically absent. We will take around 20 ml of blood per participant for complete evaluation. The major risk is during the treadmill test. In every test, a continuous electrocardiographic monitoring will be undertaken, and every two minutes the blood pressure will be measure. In case of any abnormalities during the test, it will be suspended until clarify the reason. The participant will persist or be eliminated according to the cause of the abnormality. The treadmill test will be developed inside a Hospital; therefore, any evaluation could be done immediately with the space and the equipment necessary. An emergency room will be also available during the tests. If any injury is present, we will call the specific specialist for evaluation. The costs of the evaluation and treatment will be absorbed by the Department of Endocrinology. All these risks are considered minimum because of the population that will be studied.

Expected benefits

The participant will know her clinical and biochemical results without any cost. The result of treadmill test will also be proportioned. Any abnormality will be considered for future advising after the study ends.

Risks vs. Benefits

We consider that the benefits obtained with this study are over the risks described above.

Procedure in case of injury

The participants will have the clinical researchers mobile phone which is active 24/7. In case of any complain, the participant will be invited to go to the Institution for further evaluation. The evaluation will be held by the principal investigator itself to take the decisions accordingly.

Considerations for Research in Humans

All participants who accomplished the inclusion criteria will be informed of possible benefits and risks of the study. All the procedures that will be done throughout the study will be well described and authorized by the participant. If the evaluation carries out any diagnostic abnormality, the individual will be immediately informed and orientation regarding future studies or treatments will be proportioned without cost. All clinical investigation will be conducted according to the principles expressed in the Declaration of Helsinki.

11. Costs

The research will not generate substantial cost for study participants. All the costs of clinical and biochemical evaluations, including the treadmill test will be absorbed by the Department of Endocrinology and Metabolism of the INCMNSZ.

12. References

1. **Nishimura T, Nakatake Y, Konishi M, Itoh N.** 2000 Identification of a novel FGF, FGF-21, preferentially expressed in the liver. Biochim Biophys Acta 1492: 203–206
2. **Zhang X, Yeung DC, Karpisek M, Stejskal D, Zhou ZG, Liu F, Wong RL, Chow WS, Tso AW, Lam KS, Xu A.** 2008 Serum FGF21 levels are increased in obesity and are independently associated with the metabolic syndrome in humans. Diabetes 57: 1246-53
3. **Kharitonenkov A, Shiyanova TL, Koester A, Ford AM, Micanovic R, Galbreath EJ, Sandusky GE, Hammond LJ, Moyers JS, Owens RA, Gromada J, Brozinick JT, Hawkins ED, Wroblewski VJ, Li DS, Mehrbod F, Jaskunas SR, Shanafelt AB.** 2005 FGF-21 as a novel metabolic regulator. J Clin Invest 115: 1627-1635
4. **Kharitonenkov A, Wroblewski WJ, Koester A, Chen YF, Clutinger CK, Tango XT, Hansen BC, Shanafelt AB, Etgen GJ.** 2007 The metabolic state of diabetic monkeys is regulated by FGF21. Endocrinology 148: 774-781
5. **Badman MK, Pissios P, Kennedy AR, Koukos G, Flier JS, Maratos-Flier E.** 2007 Hepatic fibroblast growth factor 21 is regulated by PPAR-alfa and is a key mediator of hepatic lipid metabolism in ketotic states. Cell Metab 5: 426-437
6. **Inagaki T, Dutchak P, Zhao G, Ding X, Gautron L, Parameswara V, Li Y, Goetz R, Mohammadi M, Esser V, Elmquist JK, Gerard RD, Burgess SX, Hammer RE, Mangelsdorf DJ, Kliewer SA.** 2007 Endocrine regulation of the fasting response by PPAR-alfa-mediated induction of FGF21. Cell Metab 5: 415-425.
7. **Cuevas-Ramos D, Almeda-Valdés P, Gómez-Pérez FJ, Meza-Arana CE, Cruz-Bautista I, Arellano-Campos O, Navarrete-López M, Aguilar-Salinas CA.** 2010 Daily physical activity, fasting glucose, uric acid, and body mass index are independent factors associated with serum fibroblast growth factor 21 levels. Eur J Endocrinol 163:469-477
8. **Chen WW, Li L, Yang GY, Li K, Qi XY, Zhu W, Tang Y, Liu H, Boden G.** 2007 Circulating FGF21 levels in normal subjects and in newly diagnose patients with type 2 diabetes mellitus. Exp Clin Endocrinol Diabetes 116: 65-68
9. **Arner P, Pettersson A, Mitchell PJ, Dunbar JD, Kharitonenkov A, Rydén M.** 2008 FGF21 attenuates lipolysis in human adipocytes – a possible link to improved insulin sensitivity. FEBS Lett 582: 1725-1730
10. **Huating Li, Yuqian Bao, Aimin Xu, Xiaoping Pan, Junxi Lu, Haiya Wu, Huijuan Lu, Kunsan Xiang, and Weiping Jia.** 2009 Serum fibroblast growth factor 21 is associated with adverse lipids profiles and γ-glutamyltransferase but not insulin sensitivity in chinese subjects. J Clin Endocrinol Metab 94;2151-2156
11. **Horton TJ, Dow S, Armstrong M, Donahoo WT.** 2009 Grater systemic lipolysis in women compared with men during moderate-dose infusion of epinephrine and/or norepinephrine. J Appl Physiol 107:200-210
12. **Hill J, Timmis A.** 2002 ABC of clinical electrocardiography. Exercise tolerance testing. Br Med J 324:1084-1087
13. **Expert Panel on Detection, Evaluation, and Treatment of High Blood Cholesterol in Adults.** 2001 Executive Summary of The Third Report of The National Cholesterol Education Program (NCEP) Expert Panel on Detection, Evaluation, and Treatment of High Blood Cholesterol in Adults (Adult Treatment Panel III). JAMA 285:2486-2497

15c. Decision

**Aproved**	**X**
**Not aproved**	
**Pending result**	
